# Validation of self-administered tests for screening for chronic pregnancy-related pelvic girdle pain

**DOI:** 10.1186/s12891-021-04103-0

**Published:** 2021-03-01

**Authors:** Monika Fagevik Olsén, Paulina Körnung, Sophie Kallin, Helen Elden, Gunilla Kjellby Wendt, Annelie Gutke

**Affiliations:** 1 Department of Health and Rehabilitation/Physiotherapy, Institute of Neuroscience and Physiology, Sahlgrenska Academy, University of Gothenburg, S-405 30 Gothenburg, Sweden; 2 Department of Physical Therapy, Sahlgrenska University Hospital, S-413 45 Gothenburg, Sweden; 3 Institute of Health and Caring Sciences, Sahlgrenska Academy, University of Gothenburg, Sahlgrenska Academy, S-405 30 Gothenburg, Sweden; 4 Department of Obstetrics and Gynecology, Institute of clinical sciences, Sahlgrenska Academy, University of Gothenburg, Gothenburg, Sweden

**Keywords:** Chronic, Pelvic girdle pain, Postpartum, Provocation tests

## Abstract

**Background:**

Many women develop pelvic girdle pain (PGP) during pregnancy and about 10% have chronic pain several years after delivery. Self-administered pain provocation tests are one way to diagnose and evaluate this pain. Their validity in post-partum women is not yet studied.

The purpose of this study was to evaluate the validity of self-administered test for assessment of chronic pregnancy-related PGP several years after delivery.

**Methods:**

Women who previously have had PGP during pregnancy and who participated in one of three RCT studies were invited to a postal follow up of symptoms including performance of self-administered tests after two, 6 or 11 years later, respectively. In total, 289 women returned the questionnaire and the test-results. Of these, a sub-group of 44 women with current PGP underwent an in-person clinical examination. Comparisons were made between test results in women with versus without PGP but also, in the sub-group, between the self-administered tests and those performed during the clinical examination.

**Results:**

Fifty-one women reported PGP affecting daily life during the last 4 weeks, and 181 reported pain when performing at least one of the tests at home. Those with chronic PGP reported more positive tests (*p* < 0.001). There was no significant difference between diagnosis from the self-administered tests compared to tests performed during the in-person clinical examination (*p* = 0.305), either for anterior or posterior PGP. There were no significant differences of the results between the tests performed self-administered vs. during the clinical examination.

**Conclusion:**

A battery of self-administered tests combined with for example additional specific questions or a pain-drawing can be used as a screening tool to diagnose chronic PGP years after delivery. However, the modified SLR test has limitations which makes its use questionable.

## Background

More than half of all pregnant women develop pelvic girdle pain (PGP) and/or lumbar pain during or after pregnancy, however, prevalence reported varies greatly depending on diagnostic criteria [1–3]. PGP is defined as pain in the area between the posterior iliac crest and the gluteal fold most common around the sacroiliac joints and/or pubic bone, sometimes with radiating pain in the thighs [3]. The cause of the pain is unclear, but hormonal and biomechanical aspects are thought to have a considerable impact [4]. During pregnancy, the high level of the hormone relaxin makes the pelvic joints more flexible in preparation for delivery. This flexibility increases the demands on muscles and ligaments and increases the risk of pain in the area when muscles do not sufficiently compensate for the increased flexibility [3].

The prevalence for chronic PGP after delivery also varies, with ranges from 8.5 to 37% and it may persist for decades [5, 6]. Women with pain in both sacroiliac (SI) joints and the pubic symphysis during pregnancy have the highest risk of developing chronic symptoms [5]. Chronic PGP has major consequences such as reduced ability to perform daily activities and work as well as decreased health-related quality of life [6, 7]. Additionally, they have more problems with sleep and higher levels of anxiety, depression, and pain-catastrophizing thoughts [6].

Predictors for chronic PGP are PGP during previous pregnancy, lumbopelvic pain prior to pregnancy and multiple positive pain provocation tests during examination [6, 8]. According to European guidelines [3] different tests must be performed to diagnose and classify PGP, such as the Posterior Pelvic Pain Provocation Test (P4), Patrick’s Faber test, Gaenslen’s Test, Modified Trendelenburg Test, and palpation of the pubic symphysis. It is also often recommended to perform the Active Straight Leg Raise Test (ASLR), but only as a functional test [3]. One limitation of these tests is that they must be performed by an examiner. Earlier studies have evaluated incidence and prevalence of PGP by solely self-reported symptom in postal questionnaires [9–11]. However, these results must be interpreted with care as there is an uncertainty about the actual location of their pain as no tests were performed. Self-administered tests have therefore been developed to be used as a screening tool in addition to questions concerning pain and functional limitation [12, 13]. A “self-administered test” refers to a test that a person can perform on her own after verbal or written instructions. Four such tests have been described for posterior PGP: modified P4, Bridging test, modified Trendelenburg test and Patrick Faber’s test; two for anterior PGP i.e., for symphyseal pain: the modified Trendelenburg test and the MAT test and two for evaluating function: ASLR and modified SLR [12, 13]. For descriptions of the tests, see below, under Methods.

These self-administered tests have been evaluated in two previous studies in pregnancy [12, 13]. In these studies, it was concluded that modified P4 and Bridging tests can be used by pregnant women to screen for posterior PGP and that the traditionally used palpation of the symphyseal joint for anterior PGP and could be replaced by pain history, pain drawing and the MAT test as palpation of the symphyseal joint is very painful [12, 13]. In the first trial [12], the pregnant women performed the self-administered tests after on-site instructions and the tests were thereafter directly repeated the traditional way, by an examiner. In the second trial, pregnant women were asked to perform the self-administered tests at home the day before the appointment with a specialized physiotherapist to assess the symptoms [13]. The results were compared to results of the tests performed during the visit. Both trials included women who visited a specialist clinic [12, 13]. To date, there are no studies concerning the validity of the self-administered tests’ in patients with chronic PGP after pregnancy.

The purpose of this study was therefore to evaluate the validity of self-administered tests for chronic pregnancy-related PGP among women with or without such pain by analyzing the consistency between the self-administered tests and subsequent diagnosis set by the self-administered tests and a questionnaire vs. a clinical examination.

## Methods

This is a part of a long-term follow-up of women with previous pregnancy-related PGP [6] who, participated in one of three randomized controlled trials evaluating different treatment modalities two, six, and 11 years previously [6]. In this follow-up 530 women were contacted [6]. The ethics committee at University of Gothenburg, Gothenburg, Sweden approved the study in May 2012 (Dnr 193–12) and the women gave their written informed consent in the questionnaire. Postal questionnaires were sent and the women who consented were asked to return the questionnaire within 2 weeks. Two reminders were sent, four and 6 weeks later respectively, if the questionnaire was not returned. The number of women responding to the survey was 371 (70%), of which 26 were excluded due to on-going pregnancy or systemic disease, leaving 345 women included in the follow-up [6].

The questionnaire included questions concerning pain and physical function. One of the questions was asked if they have had experienced lumbopelvic pain which had affected their daily life more than 1 day during the last 4 weeks, except for pain because of menstruation or fever. This question is recommended by a modified Delphi study conducted with 28 experts in back pain research from 12 countries [14]. The response of the questions was included to be able to divide the women into two groups those with and without chronic pain. The women were also asked to complete a pain drawing and perform self-administered pain provocation tests to reproduce their PGP. In addition, to be able to distinguish between lumbar pain and PGP, self-administered versions of the ASLR and the modified SLR were also added. All tests were carried out bilaterally according to written instructions and drawings/photos [12, 13]:

- Anterior PGP– a positive test had to reproduce pain in the symphyseal area
  - ○ Modified Trendelenburg test: Standing on one leg, with the other leg in 90° flexion in hip and knee.
  - ○ MAT test: Standing on one straight leg, having the opposite leg straight and the foot in constant contact with the floor as the leg is abducted to the side. This is followed by an immediate adduction back to start position, with pressure on the toes. This is simulating the movement “to pull a mat”.
- Posterior PGP- a positive test had to reproduce pain around the SI-joints
  - ○ Modified Trendelenburg test: Standing on one leg, the other in 90° flexion in hip and knee.
  - ○ 4P test (Posterior Pelvic Pain Provocation test): In the supine position with one leg in 90° hip flexion, the patient pressed with her hands on the flexed knee, along the longitudinal axis of the femur.
  - ○ Bridging test: In the supine position with bent knees and the feet on the ground, the buttocks were lifted, and one leg at a time was extended in the air until the hip and knee was in a neutral position.
  - ○ Patrick Faber’s test: In the supine position with one hip flexed, abducted, and rotated so that the heel rested on the opposite kneecap. This position should be maintained for 5 s.
- Additional tests
  - ○ Modified SLR (Straight Leg Raise test): Sitting with straight legs and 90° hip flexion. Positive test was reported if radiating pain in either of the legs.
  - ○ ASLR (Active Straight Leg Raise test): In supine position, one leg at a time was raised 20 cm from the surface. The women reported how difficult it was to raise the leg on a six-point lickert-scale from 0 (no difficulties to perform) to 5 (impossible to perform) and if it caused pain.

**Fig. 1:**
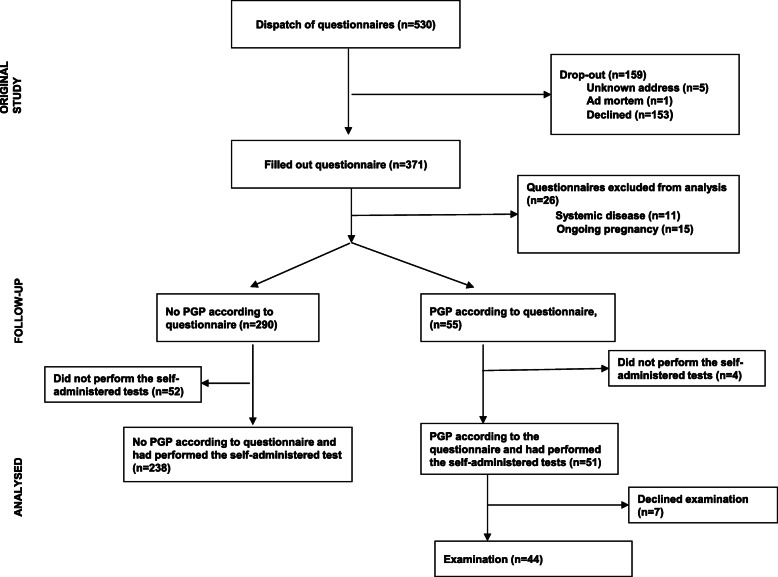
Flow-chart of the study. PGP=Pelvic Girdle Pain

Women who reported chronic PGP were offered an in-person clinical examination by a specialized physiotherapist. During the examination, the women were asked about their pain history and what provoked and eased their pain and when. They also underwent an examination to diagnose whether the pain originated from the lumbar or pelvic region or none of these areas [6]. The measuring instrument for assessing mechanical lumbar impact was Mechanical Diagnosis and Therapy [15]. The tests for PGP were the same as the self-administered tests but instructed/performed traditionally by an examiner [3, 16]. The PGP diagnosis was set according to the definition in the European guidelines: pain in the SI joints and/or pubic symphysis which had to be reproduced by specific clinical tests performed by an examiner [3, 6]. Fig 1 shows the flow-chart of the study.

The women were also diagnosed regarding PGP based on the self-administered tests and the questionnaire, according to the same criteria as during the examination [6] but adjusted concerning the tests:

- Self reported PGP during the past 4 weeks affecting daily life during a minimum of 1 day (according to the questionnaire).
- a positive pain-drawing i.e., the area of the SI-joints and/or pubic symphysis was marked.
- at least one positive self-administered test for anterior PGP i.e., symphyseal pain and / or two positive tests for posterior PGP.

Women who reported pain during at least 1 day during the last 4 weeks but had no positive pain provocation tests for the SI-joints or pubic symphysis but reported pain when performing modified SLR and /or ASLR test were classified with lumbar pain.

The consistency between self-administered tests and the tests performed during the clinical visit were analyzed by comparing the results of the tests and subsequent diagnosis in the women who underwent the in-person clinical examination.

The statistical program SPSS version 25 was used for the statistical analysis.

For analysis between the groups (PGP or not) with regards to dichotomous variables (positive and negative tests), a Chi-square test or Fisher’s exact test was used and for continuous variables a t-test was used. Sensitivity, specificity, negative predictive value (NPV) and positive predictive value (PPV) were calculated between the self-administered test and the test performed at the clinical examination.

A *p*-value < 0.05 was defined as significant.

## Results

Of the 345 women who participated in the follow-up, 56 did not perform the self-administered tests leaving 289 women who were included in this part of the study. Demographic data, in total and for the women with (*n* = 51) and without (*n* = 238) PGP is presented in Table 1. There were no significant differences between the groups.

**Table 1:** Baseline characteristics of women who 2, 6 or 11 years earlier had PGP

	Total *n* = 289	Reporting PGP *n* = 51	No PGP *n* = 238	***p***-value
**Age (years), at inclusion**	31.0 (4.3)	30.6 (3.6)	31.1 (4.4)	0.459
**Age at follow-up, years**	39.3 (5.6)	37.8 (4.6)	39.6 (5.8)	0.064
**Parity (n)**	2 (1–6)	2 (1–5)	2 (1–6)	0.741
**Index pregnancy (n)**	3 (1–5)	3 (1–4)	3 (1–5)	0.439
**Vaginal delivery (%)**	82.9	79.7	91.3	0.080
**Marital status** **Married or cohabiting/Single (%)**	91/9	91/9	91/9	0.906
**Body Mass Index (kg/m**^**2**^**)**	24.7 (4.1)	24.7 (4.3)	24.7 (4.0)	0.942
**Days of physical activity/ week (n)**	4 (0–7)	3 (0–7)	4 (0–7)	0.725

Mean (±SD) or median (min-max).

Of the 289 women, 181 reported to have at least one positive self-administered test. A positive Patrick Faber test was reported most frequently (27.7%) followed by the positive P4 test (23.5%) and Bridging test (20.8%). There were fewer positive test-results reported for tests which provoked pain in the public symphysis region.

The number of positive self-administered tests for the whole group and divided in those with or without PGP are presented in Table 2. There was a significant difference in the number of positive tests between the two groups in all included tests. In addition, the proportion of women with at least one positive test of the bilateral six pelvic pain provocation tests (number of women with a positive test(s)/total number of women in the group) was significantly higher in the group reporting PGP in the last 4 weeks than in those reporting no pain (*p* < 0.001). As only women with chronic pain fulfilled the definition to be diagnosed with PGP, no-one in the group with no pain (*n* = 238) was diagnosed with PGP. In this group 159 (67%) did not report any positive provocation tests in the symphyseal joint or sacroiliac joints. Of the remaining women in that group, one positive test was reported by 21 (9%), two positive tests by 19 (8%) and ≥ 3 positive tests by 39 women (16%).

**Table 2:** Results of positive self-administered pain provocation tests

Self-administered test	Number of positive tests							
	Total *n* = 289	PGP *n* = 51	No PGP *n* = 238	***p***-value between groups	Sensi-tivity	Speci-ficity	NPV	PPV
**Tests for anterior pgp**								
**Trendelenburg, symphyseal pain**	16 (5.5%)	9 (17.6%)	7 (2.9%)	0.001	17.6	2.9	84.6	56.3
**MAT**	24 (8.3%)	12 (23.5%)	12 (5.0%)	< 0.001	23.5	5.0	85.3	50.0
**Tests for posterior pgp**								
**Trendelenburg, posterior pain**	46 (15.9%)	25 (49.0%)	21 (8.8%)	< 0.001	49.0	8.8	84.8	54.3
**P4**	68 (23.5%)	29 (56.9%)	39 (16.3%)	< 0.001	56.9	16.4	90.0	42.6
**Bridging**	60 (20.8%)	29 (56.9%)	31 (13.0%)	< 0.001	56.9	13.0	90.4	48.3
**Patrick Faber**	80 (27.7%)	31 (60.8%)	49 (20.6%)	< 0.001	60.8	20.5	90.9	51.7
**Total number of positive PGP tests, 0–12**	0 (0–12)	4 (0–12)	0 (0–12)	< 0.001				
**Additional tests**								
**ASLR, pain**	66 (22.8%)	30 (58.8%)	36 (15.1%)	< 0.001	58.8	15.1	82.9	100
**ASLR, difficulty**	0 (0–8)	2 (0–8)	0 (0–6)	< 0.001				
**Modified SLR**	21 (7.3%)	11 (21.6%)	10 (4.2%)	< 0.001	21.6	4.2	85.1	52.4

Sensitivity: proportion of positives that are correctly identified by the test.

Specificity: proportion of negatives that are correctly identified by the test.

NPV (Negative Predictive Value): proportion of patients with a positive test who are correctly diagnosed.

PPV (Positive Predictive Value): proportion of patients with a negative test who are correctly diagnosed.

*P4* Posterior Pelvic Pain Provocation test, *ASLR* Active Straight Leg Raising test, *SLR* Straight Leg Raise test, *PGP* Pelvic Girdle Pain.

When applying the criterion of one positive test in the symphyseal joint and/or two in the sacroiliac joint, 51 women (21%) reported positive test (−s) according to the definition.

The highest sensitivity, > 55%, was seen in the P4, Bridging test, Patrick Faber’s test and ASLR and the lowest for Trendelenburg test for symphyseal pain (17.6%) and modified SLR (21%). Specificity was overall low (< 21%). NPV exceeded 80% in all tests and PPV was approximately 50% in all tests except for ASLR for pain which had a specificity of 100%.

Among the 51 women with chronic pain, 44 (86%) were clinically diagnosed as having PGP based on the criteria for PGP and positive pain provocation tests. Two women had solely anterior pain in the symphyseal joint, 30 had posterior pain in the sacroiliac joints and 12 had pain both in the anterior and posterior pelvic girdle.

The results of the tests and the subsequent diagnosis from the 44 women who performed the self-administered tests and thereafter the in-person clinical examination are included in Table 3. There were no significant differences between the groups in the proportion of positive tests. There were no significant differences between the diagnosis set after self-administered tests and questionnaire and diagnosis set at the clinical follow up in anterior PGP (0.676), posterior PGP test (*p* = 0.209) or diagnosis of PGP (*p* = 0.305).

**Table 3:** Positive tests and diagnosis of PGP by self-administered tests or tests performed during the in-person clinical examination

Test		Positive self-administered tests, ***n*** = 44	Positive tests during the in-person clinical examination, ***n*** = 44	***P***-value between the groups
**Trendelenburg, symphyseal pain**		8	12	> 0.999
**MAT-test**		11	17	0.724
**Trendelenburg, posterior pain**		21	14	0.197
**P4**		24	31	0.341
**Bridging**		22	20	0.121
**Patrick Faber**		29	13	0.164
**ASLR, difficulty**		23	26	0.650
**SLR**		8	3	0.498
**Diagnosed anterior PGP**		13	8	0.676
**Diagnosed posterior PGP**		27	27	0.209
**Diagnosis of PGP**	No pain	0	5	0.305
	Symphyseal	3	3	
	One sided SI	2	3	
	One sided SI and symphyseal	1	2	
	Bilateral SI	16	13	
	PGS	11	9	
	Back pain	11	9	

*P4* Posterior Pelvic Pain Provocation test, *ASLR* Active Straight Leg Raising test, *SLR* Straight Leg Raise test, *PGP* Pelvic Girdle Pain, *PGS* Pelvic Girdle Syndrome.

## Discussion

The main finding from this study is that there is no significant difference between the diagnosis of PGP by self-administered tests and a questionnaire versus diagnosis after an in-person clinical examination in women with chronic PGP up to 11 years after pregnancy. This was found for both posterior PGP and anterior PGP. In other words, the battery of self-administered tests is valid and can be used as a screening tool by the woman or healthcare professionals as an initial evaluation before further assessment and/or referral to treatment.

From our previous studies of the self-administered tests, we learned that the tests can already be used during pregnancy by the pregnant woman herself or by midwives or other health care professionals when pregnant women report PGP at the antenatal unit [12, 13]. An early diagnosis of PGP during pregnancy may counteract the development of increased pain and decreased function that is commonly seen during pregnancy [17]. The present study shows that the self-administered tests can support identification of chronic PGP after pregnancy as well.

Early identification of PGP is important for a woman’s health during pregnancy, as back pain including PGP is one of the most common reported barriers to being physically active in pregnancy [18, 19]. Early identification and intervention of PGP may prevent lower physical activity levels and low functioning of daily activities caused by pain, which is commonly reported by pregnant women [20]. Chronic PGP is a reality for many women in the world, and causes not only suffering for the woman herself, but also her family and society more broadly [6, 21, 22].

In general, sensitivity was higher than specificity for all pain provocation tests, which indicates that PGP was identified by the battery of self-administered tests. Since it is important to identify PGP early in its course, to prevent long term pain [6], the higher risk of false positive are to be preferred as compared to false negatives, so women with PGP are not missed.

Taking the present study and our previous studies of self-administered test into account, the P4 and the Bridging test can be recommended for identification of posterior PGP [12, 13]. Although good clinometric on the Patrick Faber’s test was seen in the present study, inconsistence was found compared to findings in our previous two studies [12, 13]. From an anatomic point of view, the test involves both hip, pelvis and lumbar spine. There is disagreement on the reliability of the Patrick Faber’s test according to European guidelines [3]. The test result may be too difficult for non-professionals to interpret and therefore we cannot recommend it as a self-administered test.

For identification of anterior PGP i.e., symphyseal pain, the MAT test showed higher sensitivity compared to the Trendelenburg test in the present study, which is in accordance with one of our previous studies [12], but not with the other [13]. More women were identified with symphyseal pain at the in-person clinical examination as compared to the self-administered tests, but the differences were not significant different. Even if one single test results may identify symphyseal pain, the test needs to be combined with other tests, additional specific questions or a pain-drawing to increase sensitivity of the diagnosis.

In our previous study on the functional self-administered ASLR [13], fewer positive tests were seen when performed as an in-patient clinical examination compared to the self-administered tests. In contrast, the present study showed no differences between the variants of the tests. Since there are discrepancies between studies, we cannot safely recommend the self-administered ASLR. This is in accordance with the conclusion stated above that it may be too hard for non-professionals to interpret the test result of ASLR [13].

The ASLR and the modified SLR used in the present study do not belong to the pelvic pain provocation tests but are included in the survey as a self-administered test to distinguish between lumbar and / or PGP as recommended [3]. The self-administered ALSR test is like the test performed at the clinical examination but the modified version of SLR is performed in a different position. The modification was utilized as we believe that the change in position reproduces the movement but is easier to perform at home. In our previous study [13], we saw that women registered positive self-administered modified SLR tests that could not be confirmed at the clinic testing. With the present study, we confirm that modified SLR is a difficult test to interpret for a non-professional. Based on the results from the present study and our previous studies, we cannot recommend the modified version used in this trial.

There are some limitations of the trial. During the previous evaluations of the self-administered tests pregnant women were included, and the tests were performed at the clinic or the day before the visit to the clinic [12, 13]. In this trial we do not know how many days before the visit the women performed the tests at home; for some women it was weeks between when they performed the self-administered tests until they were tested again during their clinic appointment. There is a risk that this delay has interfered with the results. On the other hand, the significant differences in test results between the groups, with and without PGP seems to verify that the tests are not only valid when performed during pregnancy but also in chronic PGP.

Even though the self-administered tests were performed after written instructions and photos/illustrations, there is a risk that they were incorrectly performed which is a limitation. Another possibility is to use digital platforms where the tests can be presented by short films. The tests may then be disseminated and subsequently performed wherever or whenever needed.

Future trials are needed to further evaluate the use of self-administered tests in different groups of patients for instance as screening for PGP early in pregnancy. Use of different instruction methods also need to be investigated to find the optimal way to describe how to perform the test in a correct way.

This study is the first to present validation of the self-administered tests to diagnose chronic PGP. Based on the results from previous studies of the self-administered tests, there is now a good reason to use these tests in clinical practice during and after pregnancy [6, 12, 13].

## Conclusion

A battery of self-administered tests combined with for example additional specific questions or a pain-drawing can be used as a screening tool to diagnose chronic PGP years after delivery. However, the modified SLR test has limitations which makes its use questionable.

## Data Availability

The datasets used and analyzed during the current study are available from the corresponding author on reasonable request.
